# Political and Cultural Foundations of Long-term Care Reform: Comment on "Financing Long-term Care: Lessons From Japan"

**DOI:** 10.15171/ijhpm.2019.90

**Published:** 2019-10-28

**Authors:** Ming-Jui Yeh

**Affiliations:** Department of Health Policy and Management, Emory University, Atlanta, GA, USA.

**Keywords:** Democracy, Intergenerational Solidarity, Confucian Ethics, Responsibility for Care, East Asia

## Abstract

This paper comments on Naoki Ikegami’s editorial entitled "Financing long-term care: lessons from Japan." Adding to the editorial, this paper focuses on analyzing the political and cultural foundations of long-term care (LTC) reform. Intergenerational solidarity and inclusive, prudential public deliberation are needed for the establishment or reform of LTC systems. Among various lines of ethical reasoning related to LTC, Confucian ethics and other familist ethics are specifically important in the societies that share these values. The core issue in the debates around LTC reform is how to (re-)define the scope of social entitlements and accordingly to allocate the responsibility for care between states and families, between social groups, and between generations with limited resources.

In a recent editorial,^1^ Ikegami proposes experiences related to financing the Japanese long-term care (LTC) system. Based on the LTC insurance (LTCI) established in Japan in 2000, Ikegami suggests that, for countries considering establishing a publicly-funded LTC system, it is better to initiate the project at an early stage when LTC services have not yet been covered by other systems (the health system, in Japan’s case) or perceived as part of the entitlement of citizenship, because these services promised by politicians would later become an unbearable burden on the LTC system. In such cases, any further reform and cost containment attempts (eg, increased contribution rate, revised fee schedule, reduced benefits, and stricter eligibility criteria) would have only marginal effects.

As the earliest social insurance for LTC in Asia, Japan’s experiences offer revealing information for other countries to scrutinize. The LTCI alleviates the social care burden of the health system, most notably the “social beds” set aside in hospitals, and instead meets these care needs with the less costly LTC services. Despite its increasing expenditure, the LTCI also provides universal coverage for LTC needs for all Japanese residents. Supposing that universal coverage is a common good to be achieved by a society, the LTCI in Japan is indeed a successful case. South Korea also adopted a social insurance scheme for LTC in 2008.^2^ Policy-makers in Taiwan made a similar attempt in 2016, but the proposal was substituted with a tax-based model.^3^ Adding to the editorial, this paper analyzes several contextual political and cultural factors of LTC financing as well as broader reform issues that are also worth noting through a comparative perspective^4^ for further discussion.

## The Political Foundations of Long-term Care Reform

While Ikegami’s suggestion to establish an LTC system at an early stage seems reasonable and practical, the overly generous (as suggested by Ikegami) care responsibility of the LTCI is also the logical result of democratic accountability. If the politicians who promised the people they would publicly fund LTC services were able to win elections, it suggests that financing LTC indeed reflects the will of the people, and that unmet LTC needs have become an important issue for citizens. The LTCI, then, was the formalization of this demand as the politicians were held accountable by the people.

If the path-dependent LTC system representing an overly generous service package is a problem, it is a problem with democratic politics rather than LTCI *per se* . For instance, one could rightly argue that electoral democracy is short-sighted in nature; people tend to make decisions based on their short-term self interests (receiving as much reimbursement from the LTCI as possible to mitigate their financial burden) rather than the common good of the political community in the long run (the financial sustainability of the LTCI system).^5^ This phenomenon has been called “presentism” by political scientists.^6^ The democratic decisions would therefore tend to be biased toward present generations, putting more responsibility/burden on future ones (including not-yet-born future citizens and citizens under legal age to exercise political rights) ^[1],7^ hence making the LTC system overly generous and fiscally irresponsible (to adopt Ikegami’s term).

Under this circumstance, policy-makers and reformers could take into consideration the notion of intergenerational solidarity and prudential public deliberation. Intergenerational solidarity could justify the present-biased policy arrangements in that it presumes the obligation of mutual assistance between present and future generations.^9^ Suppose intergenerational solidarity exists; then future generations would be willing to inherit the (likely financially unsustainable) LTCI. If this is the case, policy-makers need not be concerned about the financial issues of the LTC system, nor about the present-biased problem. However, this approach has an obvious limitation in that the present generation cannot possibly know the actual preference of future ones; hence whether intergenerational solidarity exists would not be verifiable. One way to get past this limitation is to ignore it and simply presume that intergenerational solidarity does exist. However, if we find this presumption too strong, another way is to rely on a more inclusive and prudential public deliberation process.

Prudential means that the deliberation is based on reasoning, specifically the deliberators’ self-restraint from over-exploiting resources in the future. Inclusive means that the deliberation is open to every social group at stake; all those who would be affected by the policy arrangements should have a voice in the deliberation process.^10^ Through such public deliberation, the people could better clarify and further redefine the essence of entitlement of citizenship that should be publicly funded by a LTC system, such as the LTCI in Japan.

Like Ikegami has suggested, initiating the LTC project at an early stage could not only prevent the system from inheriting the care burden from previously-committed entitlements and hence be “fiscally more responsible” (p. 465), it could also allow the policy-makers and stakeholders sufficient time to go through the prudential public deliberation process, and hence better tackle the present-biased problem of democratic decisions. Nevertheless, this strategy has limitations as well.

First, prudence is indeed an abstract concept not easily operationalized. A recent report on asset management in the aged society released by the Financial Services Agency of the Japanese Government in June 2019 is an example. The report estimates that a couple in Japan, expected to live to 95 years old, will need to set aside 20 million yen (about 190 000 US dollar) in pensions for their retirement life.^11^ While this report aroused much public debate and unease, it is in a sense a practical and realistic warning. However, heavy criticism from the people eventually forced the government to retract the report. This result suggests that prudential estimations made by elite bureaucrats, not even to the extent of deliberation, are often not cordial to popular sentiment. Despite these limitations, the examples of public deliberation of health reforms in Taiwan show that while the participants could not offer concrete policy indications, they were empowered by the process and their attitudes and knowledge toward policy were changeable.^12,13^ A study in Japan also shows similar potential of deliberation to empower intergenerational thinking.^14^

Second, initiating the LTC project at an early stage implies that unmet LTC needs have not yet become a major issue among the people. Consequently, neither do politicians have the pressures/incentives to initiate the project, nor are enough people interested in or satisfied with such proposals. Other, more relevant issues would occupy the policy agenda. In such cases, policy-makers and reformers probably need a policy window,^15^ such as a series of tragic events or the political will of a powerful leader (or a dictator in a non-democratic or democratizing context),^16^ to trigger the establishment/reform of an LTC system.

In a democratic polity, present-biased decisions are embedded in popular demands. Prudential public deliberation may be available as a supplementary source of democratic accountability and information regarding the public will for policy reformers to consider. Nevertheless, policy-making is still largely dependent on the balance of political forces and other contingent factors. Reformers should take this nature into account and seize the chances when they appear.

## The Cultural Foundations of Long-term Care Reform

Besides the political environment, other contextual factors also enable or impede the reform of LTC systems. Ikegami’s observation on the family care burden, specifically the implicit (formerly legal in Japan) obligations of wives and daughters-in-law, is straightforward and yet quite precise, as filial piety and patriarchism are still the core values upheld by most East Asian societies that are more or less affected by Confucian ethics.^17,18^

The conflicting values between universal citizenship and the traditional Confucian care model would bring cultural tension to LTC reforms.^19^ In universal citizenship, which is the model adopted by most publicly-funded LTC systems, citizens are equally eligible to receive LTC services based on their needs; while in the Confucian model, the responsibility for care is differential: it is first the responsibility of direct family members (eg, adult children of older parents in need) and second of other relatives (eg, siblings, adult children of siblings); lastly, public money only pays for those who are left outside the familial network or are in poverty. These two opposite normative values on the allocation of responsibility for care should be addressed by policy reformers.

On one hand, if reformers intend to build a more universal LTC system with comprehensive coverage, responsibility for LTC would be more generalized to the whole society. This approach would require more public funds to support the system, as seen in the LTCI in Japan. For policy-makers in other countries that are just about to initiate an LTC project, this approach could tackle the unfair allocation of LTC responsibility to women through formalized mutual assistance. South Korea is a case representing this approach, as the country adopted a universal LTCI like Japan’s and formally shifted the responsibility for care from families to the publicly-funded system; LTC services have become a right entitled to citizens.^2^ However, for reformers wrestling with financial concerns, the expansion toward universal LTC coverage is clearly not a viable option as it would require more public money input.

On the other hand, if policy reformers intend to refrain from welfare expansion in LTC and just build a supplementary LTC system grounded in the traditional Confucian care model, they would inevitably reproduce and, even worse, formalize the inherent oppression of women. Yet shifting responsibility for LTC back to families in the name of traditional values could be a palatable option for those who wish to save public expenditures and adopt “restricted universalism”^20^ for consideration. The tax-based LTC system in Taiwan could be seen as a case of this supplementary approach. While nominally all citizens in need are entitled to subsidies – making the system appear universal – it could only subsidize supplementary LTC services for families without adequate care capacity or funding.^9^ This arrangement reflects the values of differential responsibility under Confucian ethics. It represents not only the concerns of Confucian ethics, but also other conservative cultures with familial ethics.^21^

Social insurance schemes and tax-based models are of course not dichotomous options, but different financing mechanisms. Other private-public mixtures of LTC financing, like the savings accounts in Singapore, could also be considered.^22^ The point is that the cultural tension between the formal system and traditional values should be the concern of countries where societies share these familial values.

## Reasonable Allocation of the Responsibility for Care

In the classic scene in Imamura Shohei’s 1983 film *The Ballad of Narayama* (Narayama bushiko), Tatsuhei could not help but ran back to where he put down his mother on the hill of Nara Mountain and yell, “Mom, it’s snowing (oka, yuki ga futte kida yo)!” to his mother at a distance ^[2] ^.^23^ Whether this practice of abandoning old parents when they have lived to the proper age (*obasute* ) is a real custom or merely a legend is subject to anthropological investigation, but the lesson from this story is that every society has to evolve in a balanced way to allocate responsibility for care under limited resources.^26^

While publicly-funded LTC systems are very different from the LTC arrangements in rural villages in the early-era Japan in Imamura’s film, this core issue remains the same today. The prospect for financing LTC might not be such a grey area as people thought,^27^ but difficult choices still need to be made^28^; the difference is just that they may be more subtle and implicit in this era. One could of course demand more public resources to be allocated to LTC services by drawing on the notion of human rights or other ethical grounds^29^; however, this implies that the same amount of resources cannot be allocated for other social services that are also considered essential entitlements of citizenship.

To make these difficult choices, either stronger intergenerational solidarity or better-designed public deliberation is needed. Further, for ethical reasoning to be grounded in societal values, Confucian ethics as well as other familist ethics should be taken into account because they are interwoven with LTC arrangements. The eventual task is to define or redefine the scope of social entitlements and hence the reasonable allocation of responsibility for care between the state and families, between social groups, and between generations^ [3] ^.

Japan has always been a pioneer in the development of health and social systems in East Asia. Grounded in strong popular support as well as path-dependent generous entitlements, Japan managed to introduce a universal LTCI in 2000. Its lessons as offered by Ikegami are not to be overlooked by policy researchers and reformers of middle-income countries in this region and other countries seeking solutions for mitigating the care burden of families as well as the augmented financial burden of the LTC sector.

## Ethical issues

Not applicable.

## Competing interests

Author declares that he has no competing interests.

## Author’s contribution

MJY is the single author of the paper.

## Endnotes


[1] However, some psychological studies have suggested an interestingly opposite decision preference when people are other-regarding (instead of self-regarding) under an experimental context.^8^



[2] The villagers believed that if it snows on the days the elder “go to the Mountain,” it means good fortune; hence, Tatsuhei was earnest to share this news with his mother, while he was not supposed to turn back once he had left her (hence the self-refraining distance). The Ballad of Narayama has become a classic text for the discussion of respect to life and morality of aging.^24,25^



[3] As one reviewer rightly suggested, the market also plays an important role in the debate around LTC reform. However, from the perspective of financing, the state, families, or individuals (in different social groups) could all be financial agents who actually pay for LTC services; but the market cannot. Market might be a mechanism to allocate funds or to deliver services, but a market itself cannot and will not be responsible for paying for anyone’s care needs.

